# Supplementary material for: Impact of Duodenal Stump Reinforcement in Preventing Duodenal Stump Fistula/Leakage After Distal or Total Gastrectomy for Malignant Disease: A Meta-Analysis of Comparative Studies
Source: Cancers (Basel). 2025 May 22;17(11):1735. doi: 10.3390/cancers17111735 (PMC12153669; doi:10.3390/cancers17111735)
Supplement: Supplementary file 1 [file cancers-17-01735-s001.zip › cancers-3597632-supplementary.pdf]

Supplementary material

Table S1. DSF definitions.

Authors/Year	DSF definition
Sano et al/2024 [31]	Presence of duodenal juice in the surgical drainage or its leakage through the abdominal wall or confirmed based on computed tomography, fistulography, and/or surgical findings if performed
Wang et al/2024 [22]	Presence of duodenal fluid in the surgical drainage. Moreover, patients who exhibit clinical suspicious symptoms and signs of DSF such as sudden and severe abdominal pain, tenderness, and rebound tenderness were confirmed by radiological findings using abdominalpelvic computed tomography scan or fistulography.
Sun et al/2024 [21]	Leakage occurring at the duodenal stump after surgery, leading to the leakage of digestive fluid or other gastrointestinal contents into the abdominal cavity or surrounding tissues. The diagnosis of duodenal stump leakage is based on one or more of the following: 1) Clinical evidence of biliary drainage in the drain tube or abdominal wound, without other evidence of anastomotic leakage. 2) Computed tomography scan showing bile collection around the duodenal stump, followed by bile-stained aspiration or drainage. 3) Duodenography showing extravasation of contrast agent or duodenal fistula on contrast-enhanced imaging. 4) Intraoperative identification of duodenal leakage during surgical exploration.
Gu et al/2020 [30]	Presence of fluid in the surgical abdominal drain or after radiological drainage with at least 3 times higher bilirubin or amylase concentrations than normal serum value; or leakage through the abdominal wall regardless of its clinical impact confirmed by abdomen computed tomography and/or fistulography.
Ri et al/2019 [19]	n/a
Inoue et al/2016 [16]	n/a

DSF = Duodenal stump fistula; n/a = not available

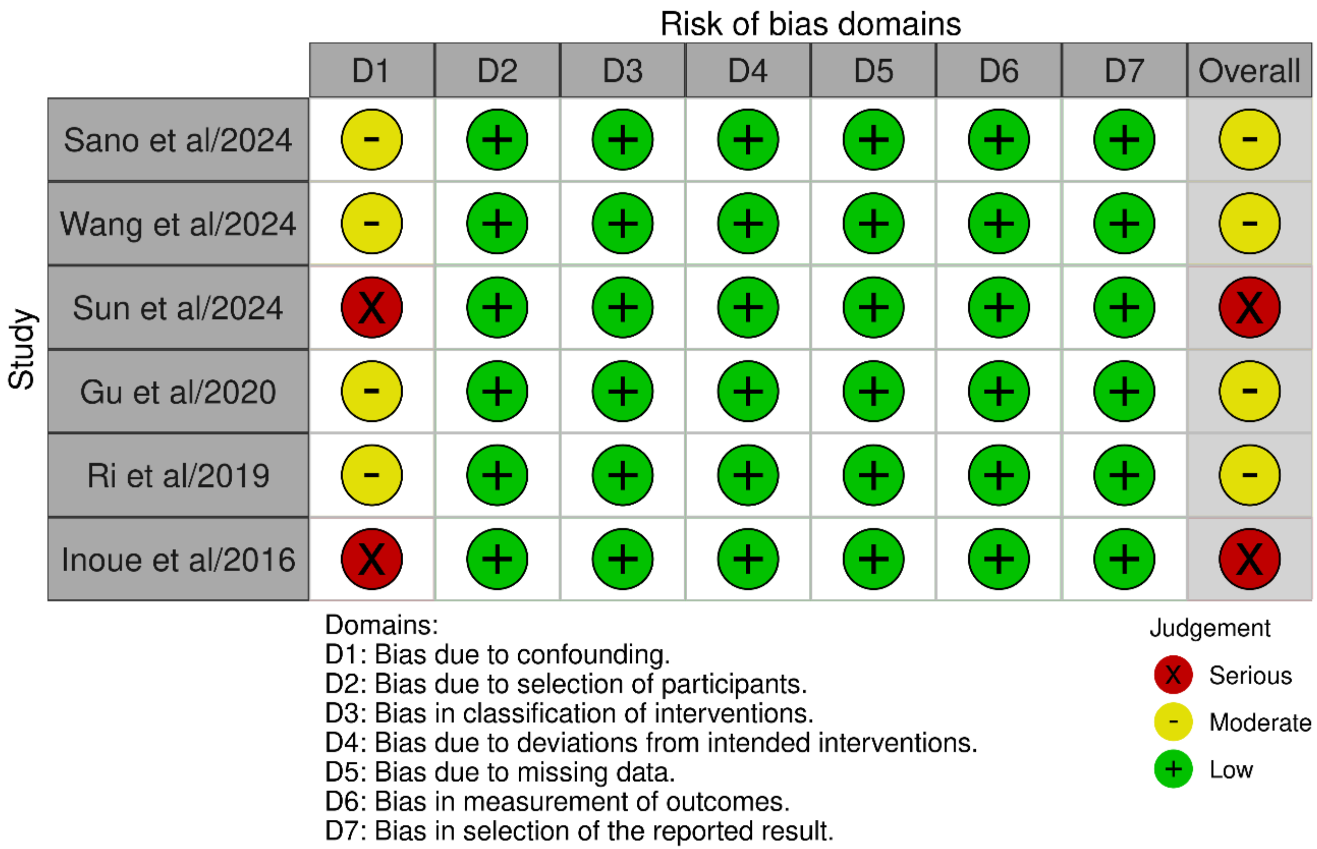
Risk of bias domains
D1
D2
D3
D4
D5
D6
D7
Overall
Study
Sano et al/2024
Wang et al/2024
Sun et al/2024
Gu et al/2020
Ri et al/2019
Inoue et al/2016
Domains:
D1: Bias due to confounding.
D2: Bias due to selection of participants.
D3: Bias in classification of interventions.
D4: Bias due to deviations from intended interventions.
D5: Bias due to missing data.
D6: Bias in measurement of outcomes.
D7: Bias in selection of the reported result.
Judgement
Serious
Moderate
Low

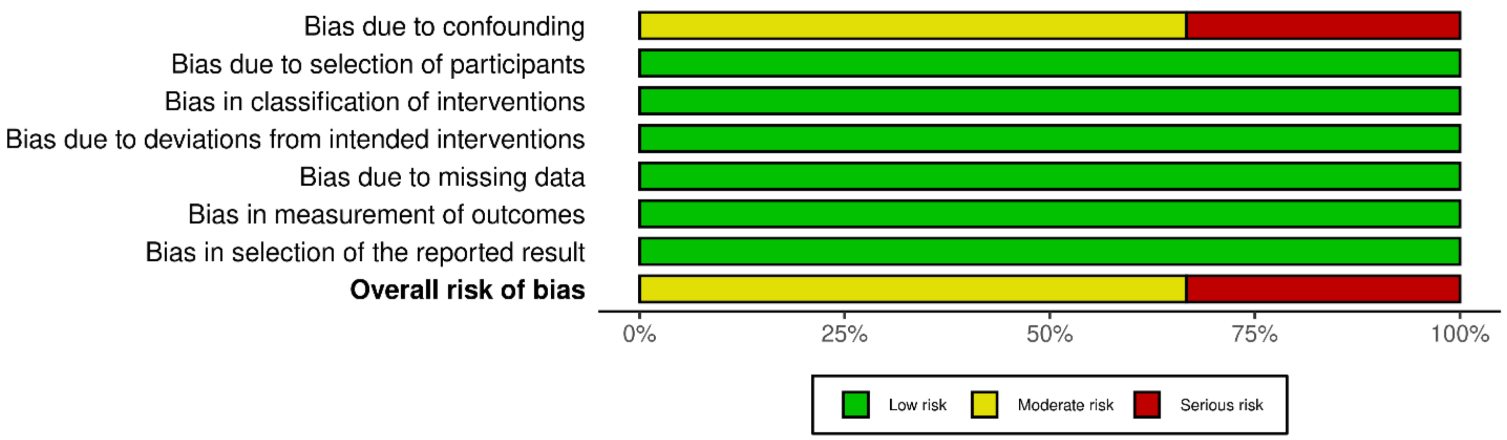

Figure S1. Retrospective studies evaluated using ROBINS-I V2.

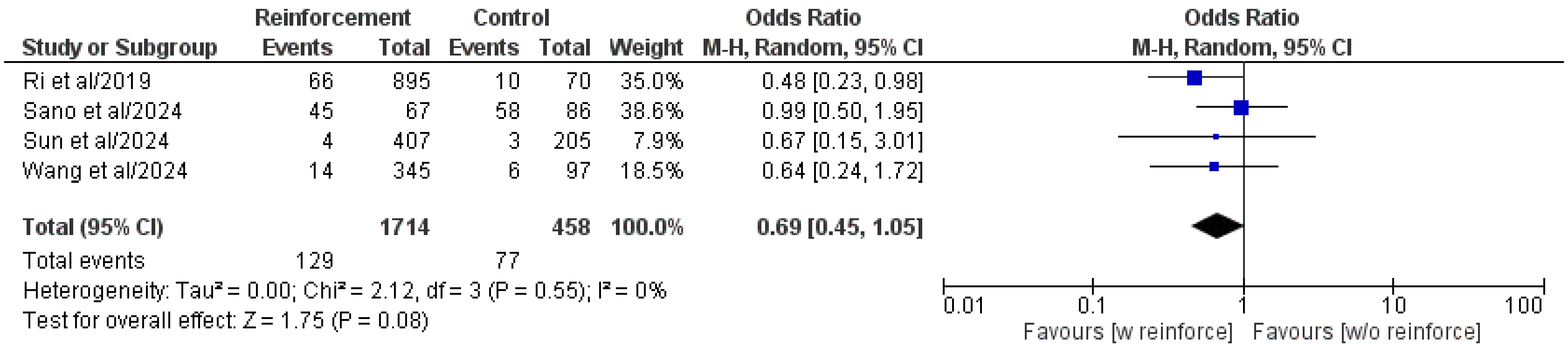

Figure S2. Forest plot comparing **major postoperative complications** between the Reinforcement and Control groups [*Leave-one-out* sensitivity analysis – **Inoue et al.**]. CI, confidence interval; M-H, Mantel–Haenszel. [19,21,22,31]

Study or Subgroup	Reinforcement Events	Reinforcement Total	Control Events	Control Total	Weight	Odds Ratio M-H, Random, 95% CI
Inoue et al/2016	2	121	5	102	9.1%	0.33 [0.06, 1.72]
Sano et al/2024	45	67	58	86	54.0%	0.99 [0.50, 1.95]
Sun et al/2024	4	407	3	205	11.0%	0.67 [0.15, 3.01]
Wang et al/2024	14	345	6	97	25.9%	0.64 [0.24, 1.72]
**Total (95% CI)**		**940**		**490**	**100.0%**	**0.77 [0.46, 1.26]**
Total events	65		72			

Heterogeneity: Tau² = 0.00; Chi² = 1.71, df = 3 (P = 0.64); I² = 0%
Test for overall effect: Z = 1.05 (P = 0.29)

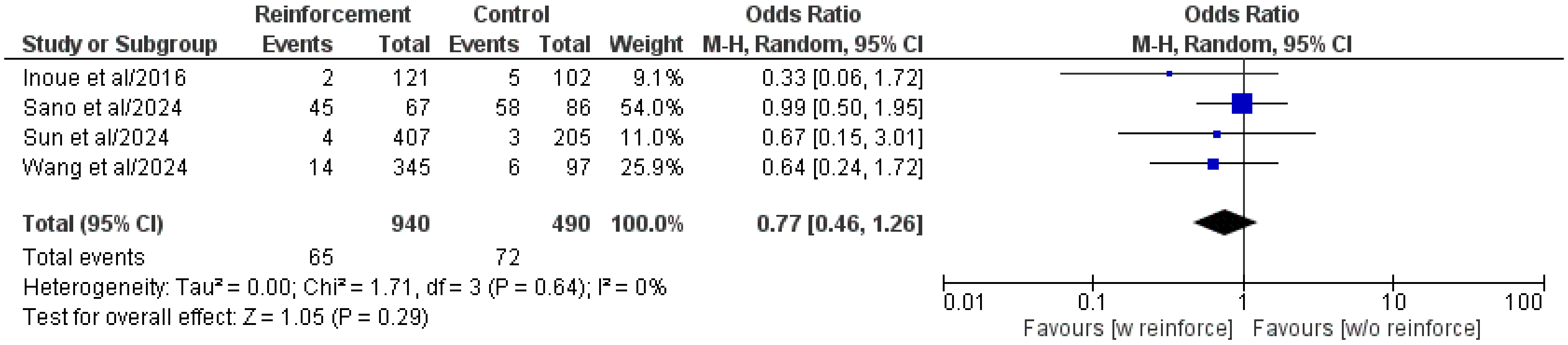

Figure S3. Forest plot comparing **major postoperative complications** between the Reinforcement and Control groups [*Leave-one-out* sensitivity analysis – **Ri et al.**]. CI, confidence interval; M-H, Mantel–Haenszel. [16,21,22,31]

Study or Subgroup	Reinforcement Events	Reinforcement Total	Control Events	Control Total	Weight	Odds Ratio M-H, Random, 95% CI
Inoue et al/2016	2	121	5	102	6.6%	0.33 [0.06, 1.72]
Ri et al/2019	66	895	10	70	35.5%	0.48 [0.23, 0.98]
Sano et al/2024	45	67	58	86	39.2%	0.99 [0.50, 1.95]
Wang et al/2024	14	345	6	97	18.7%	0.64 [0.24, 1.72]
**Total (95% CI)**		**1428**		**355**	**100.0%**	**0.65 [0.43, 1.00]**
Total events	127		79			

Heterogeneity: Tau² = 0.00; Chi² = 2.83, df = 3 (P = 0.42); I² = 0%
Test for overall effect: Z = 1.95 (P = 0.05)

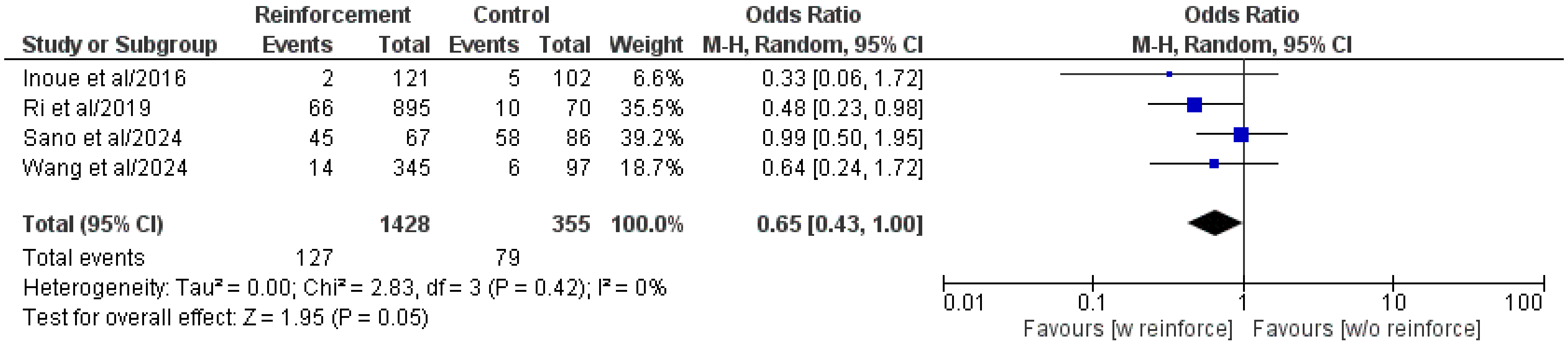

Figure S4. Forest plot comparing **major postoperative complications** between the Reinforcement and Control groups *[Leave-one-out* sensitivity analysis – **Sun et al.**]. CI, confidence interval; M-H, Mantel–Haenszel. [16,19,22,31]

Study or Subgroup	Reinforcement (SRS) Events	Total	Control Events	Total	Weight	Odds Ratio M-H, Random, 95% CI
Gu et al/2020	3	527	8	283	13.4%	0.20 [0.05, 0.75]
Inoue et al/2016	0	121	2	102	3.4%	0.17 [0.01, 3.49]
Ri et al/2019	6	895	4	70	14.0%	0.11 [0.03, 0.40]
Sano et al/2024	54	8065	86	7206	39.3%	0.56 [0.40, 0.79]
Sun et al/2024	3	407	5	205	12.0%	0.30 [0.07, 1.26]
Wang et al/2024	9	336	6	91	18.1%	0.39 [0.14, 1.13]
**Total (95% CI)**		**10351**		**7957**	**100.0%**	**0.32 [0.18, 0.58]**
Total events	75		111			

Heterogeneity: Tau² = 0.19; Chi² = 8.34, df = 5 (P = 0.14); I² = 40%
Test for overall effect: Z = 3.81 (P = 0.0001)

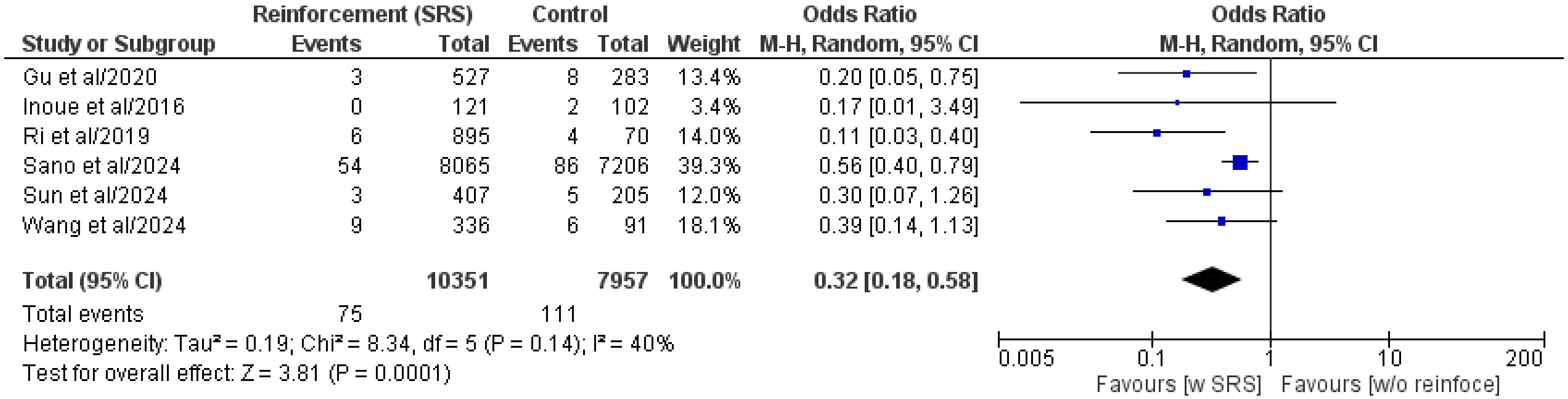

Figure S5. Forest plot comparing **duodenal stump fistula rate** between the Reinforcement (duodenal stump reinforcement with seromuscular suture of any type) and Control groups [Subgroup analysis]. CI, confidence interval; M-H, Mantel–Haenszel. [16,19,21,22,30,31]

Study or Subgroup	Reinforcement (SRS-IS) Events	Total	Control Events	Total	Weight	Odds Ratio M-H, Random, 95% CI
Inoue et al/2016	0	121	2	102	10.0%	0.17 [0.01, 3.49]
Ri et al/2019	6	895	4	70	55.9%	0.11 [0.03, 0.40]
Sun et al/2024	2	175	5	205	34.1%	0.46 [0.09, 2.41]
**Total (95% CI)**		**1191**		**377**	**100.0%**	**0.19 [0.07, 0.49]**
Total events	8		11			

Heterogeneity: Tau² = 0.00; Chi² = 1.95, df = 2 (P = 0.38); I² = 0%
Test for overall effect: Z = 3.39 (P = 0.0007)

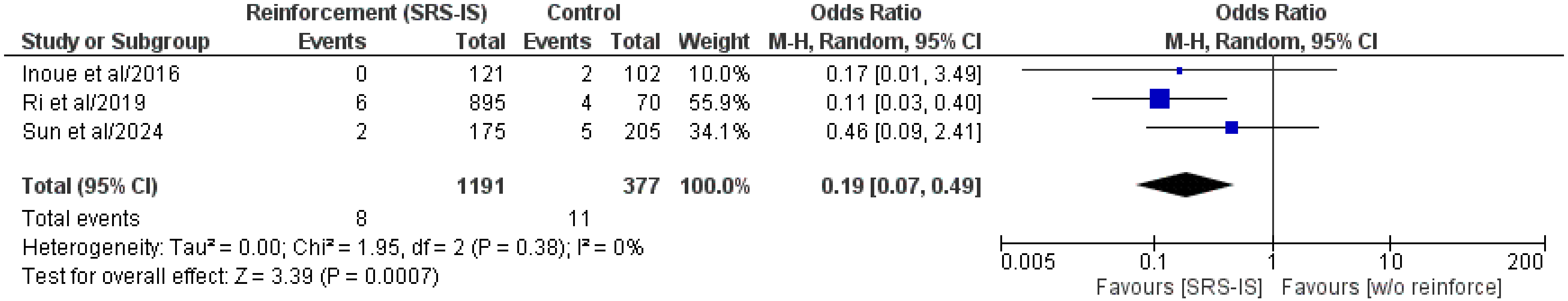

Figure S6. Forest plot comparing **duodenal stump fistula rate** between the Reinforcement (duodenal stump reinforcement with seromuscular interrupted suture) and Control groups [Subgroup analysis]. CI, confidence interval; M-H, Mantel–Haenszel. [16,19,21]

Study or Subgroup	Reinforcement Events	Reinforcement Total	Control Events	Control Total	Weight	Odds Ratio M-H, Random, 95% CI
Gu et al/2020	3	527	8	283	21.2%	0.20 [0.05, 0.75]
Inoue et al/2016	0	121	2	102	4.1%	0.17 [0.01, 3.49]
Ri et al/2019	6	895	4	70	22.8%	0.11 [0.03, 0.40]
Sun et al/2024	3	407	5	205	18.2%	0.30 [0.07, 1.26]
Wang et al/2024	9	336	6	91	33.7%	0.39 [0.14, 1.13]
**Total (95% CI)**		**2286**		**751**	**100.0%**	**0.23 [0.13, 0.43]**
Total events	21		25			

Heterogeneity: Tau² = 0.00; Chi² = 2.41, df = 4 (P = 0.66); I² = 0%

Test for overall effect: Z = 4.64 (P < 0.00001)

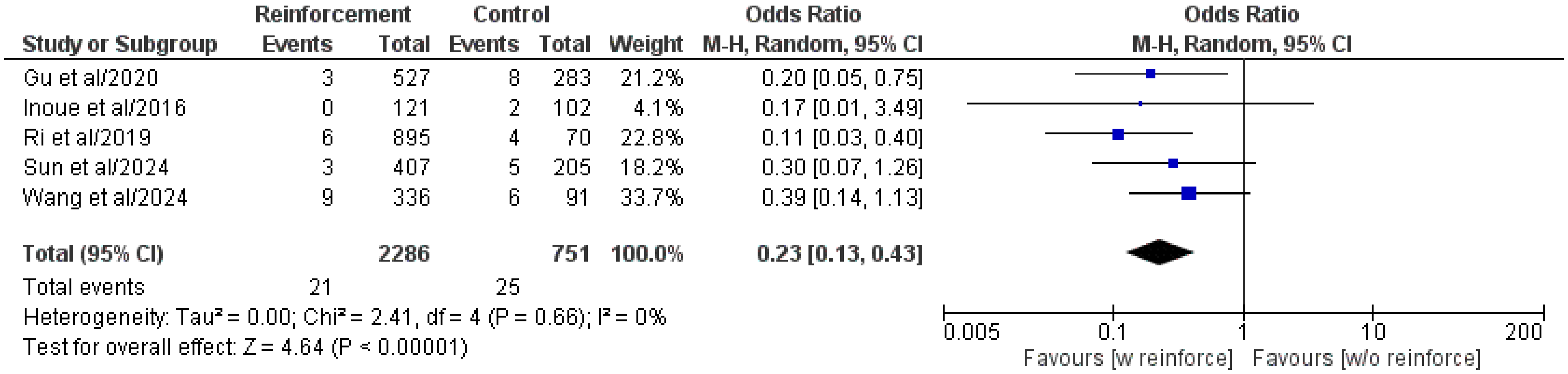

Figure S7. Forest plot comparing **duodenal stump fistula rate** between the Reinforcement and Control groups [Subgroup analysis]. CI, confidence interval; M-H, Mantel–Haenszel. [16,19,21,22,30]

Study or Subgroup	Reinforcement (SRS) Events	Reinforcement (SRS) Total	Control Events	Control Total	Weight	Odds Ratio M-H, Random, 95% CI
Gu et al/2020	3	527	8	283	21.2%	0.20 [0.05, 0.75]
Inoue et al/2016	0	121	2	102	4.1%	0.17 [0.01, 3.49]
Ri et al/2019	6	895	4	70	22.8%	0.11 [0.03, 0.40]
Sun et al/2024	3	407	5	205	18.2%	0.30 [0.07, 1.26]
Wang et al/2024	9	336	6	91	33.7%	0.39 [0.14, 1.13]
**Total (95% CI)**		**2286**		**751**	**100.0%**	**0.23 [0.13, 0.43]**
Total events	21		25			

Heterogeneity: Tau² = 0.00; Chi² = 2.41, df = 4 (P = 0.66); I² = 0%

Test for overall effect: Z = 4.64 (P < 0.00001)

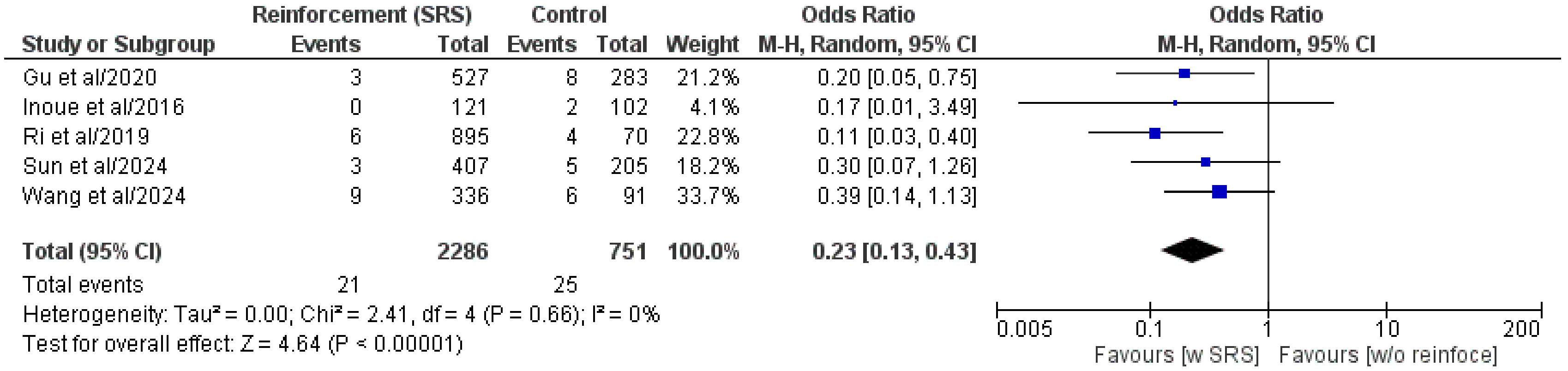

Figure S8. Forest plot comparing **duodenal stump fistula rate** between the Reinforcement (duodenal stump reinforcement with seromuscular suture of any type) and Control groups [Subgroup analysis]. CI, confidence interval; M-H, Mantel–Haenszel. [16,19,21,22,30]

Study or Subgroup	Reinforcement (SRS-IS) Events	Total	Control Events	Total	Weight	Odds Ratio M-H, Random, 95% CI	Odds Ratio M-H, Random, 95% CI
Inoue et al/2016	0	121	2	102	10.0%	0.17 [0.01, 3.49]	
Ri et al/2019	6	895	4	70	55.9%	0.11 [0.03, 0.40]	
Sun et al/2024	2	175	5	205	34.1%	0.46 [0.09, 2.41]	
**Total (95% CI)**		**1191**		**377**	**100.0%**	**0.19 [0.07, 0.49]**	
Total events	8		11				

Heterogeneity: Tau² = 0.00; Chi² = 1.95, df = 2 (P = 0.38); I² = 0%
Test for overall effect: Z = 3.39 (P = 0.0007)

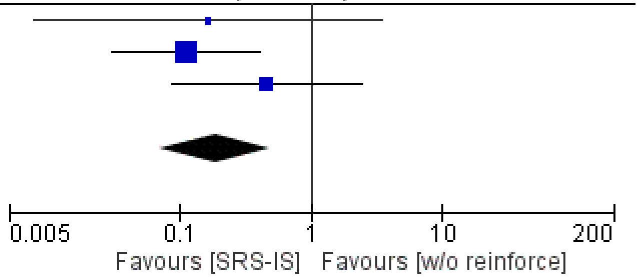

Figure S9. Forest plot comparing **duodenal stump fistula rate** between the Reinforcement (duodenal stump reinforcement with seromuscular interrupted suture) and Control groups [Subgroup analysis]. CI, confidence interval; M-H, Mantel–Haenszel. [16,19,21]
